# Defensive medicine in Danish general practice. Types of defensive actions and reasons for practicing defensively

**DOI:** 10.1080/02813432.2021.1970945

**Published:** 2021-08-31

**Authors:** Merethe K. Andersen, Elisabeth Assing Hvidt, Kjeld M. Pedersen, Jesper Lykkegaard, Frans B. Waldorff, Anders P. Munck, Line B. Pedersen

**Affiliations:** a Research Unit of General Practice, Department of Public Health, University of Southern Denmark, Odense, Denmark; b Department for the Study of Culture, University of Southern Denmark, Odense, Denmark; c Department of Business and Economics, University of Southern Denmark, Odense, Denmark; d Research Unit for General Practice and Section of General Practice, Department of Public Health, University of Copenhagen, Copenhagen, Denmark; e DaCHE – Danish Centre for Health Economics, Department of Public Health, University of Southern Denmark, Odense, Denmark

**Keywords:** General practice, defensive actions, occurrence, types, reasons

## Abstract

**Objective:**

To examine the occurrence of and types of defensive medicine (DM), and the reasons for practicing DM in general practice.

**Design:**

Prospective survey registration of consecutive consultations regarding defensive medicine defined as: Actions that are not professionally well founded but are carried out due to demands and pressure. The GPs registered the degree of defensiveness, the type(s) of defensive action(s) and the reason(s) for acting defensively.

**Setting:**

Danish general practice.

**Subjects:**

A total of 26 GPs registered a total of 1,758 consultations.

**Main outcome measures:**

Defensive medical actions.

**Results:**

Defensive actions were performed in 12% (210/1749) of all consultations. A fifth (46/210) of the defensive actions were characterised by the GPs as ‘moderately’ or ‘highly’ defensive. Frequent types of defensive actions were: blood tests, point-of-care-tests (POCTs) and referrals. Common reasons for defensive actions were: Influence from patients, 37% (78/210), concerns of overlooking severe disease, 32% (67/210) and influence from patient relatives, 12% (25/210).

**Conclusion:**

Danish GPs registered self-perceived defensive actions in a prospective survey. DM was carried out in one out of eight consultations, most often due to patient influence. The most frequent defensive actions were blood tests, POCTs and referrals.

## Introduction

Physicians are often met with expectations for certain actions that are not deemed medically warranted, but where they succumb to a perceived pressure resulting in DM [1,2]. Increased public knowledge of diseases along with advances in diagnostic and treatment options, may have led to rising expectations to the overall healthcare system by society, patients and their relatives and may explain some of the mechanisms behind DM [2–4].

Several definitions of DM have been proposed [5–7], in particular in US originated research, all of which have in common that the motivation for practicing DM is the avoidance of malpractice liability and claims reflecting the US health care set-up. This defining element was, however, challenged already in 1994 by Dutch researchers who argued that a wider conceptualization should be included in countries with little malpractice litigation [8] or no-fault malpractice such as the Netherlands and other Northern European countries along with New Zealand. Recently, a qualitative study from Danish general practice supported this wider conceptualization by proposing that DM may be defined as ‘Actions that are not professionally well founded, but are carried out due to demands and pressure’ [2].

Reported occurrence of DM varies depending on the definition, time period, and method used. Some studies report defensive actions amounting to approximately 10% of all medical actions in hospital settings [9,10]. In a questionnaire study among UK hospital doctors, 78% of respondents reported practicing one form or another of DM, ordering unnecessary tests as the most common form of DM, followed by referral to other specialties [11]. In an Italian study surveying GPs, 78% of interviewed GPs declared that they had practiced at least one form of DM during the previous working month [12]. Both studies were retrospective and thus raise concern of recall bias. The objectives of the present study are to examine the occurrence and types of DM, and the reasons for practicing DM by means of a prospective survey registration by GPs.

## Methods

### Sampling

All 785 GPs practicing in the Region of Southern Denmark were invited to participate in a prospective survey registration during a five-week period in February/March 2019.

### Patient and public involvement statement

The study does not actively involve patients, as patients did not provide information or biological materials. Neither did they receive any intervention. Hence, patient consent was not collected. GPs gave written consent to the use of their survey data for research and quality development prior to participation.

### Registration survey

The registration survey was developed based on six qualitative focus group interviews with 22 GPs [2]. In the survey, the GPs were asked to register all consecutive consultations during a period of minimum three and maximum five working days, indicating for every consultation if some form of defensive action was carried out. If DM was practiced in the consultation, GPs were asked to assess the degree of defensiveness, report the type(s) of defensive action(s) undertaken and the reason(s) for doing so. If no defensive actions were reported, the registration was finalised.

The survey further consisted of the following themes: Time of day for the consultation, patient gender, patient age, and reason for encounter according to ICPC-2-R (International Classification of Primary Care, Second edition) [13] (see Table 2 and Appendix A for further details).

The applied understanding of DM within this study context: ‘Actions that are not professionally well founded, but are carried out due to demands and pressure’, emerge from the qualitative study [2], and was printed on top of the registration survey. The GPs were further introduced to this understanding in the enclosed instruction.

### Background survey

In addition to the registration survey, the GPs completed a survey with background information about: GP’s age and gender, years in practice and practice type.

### Pilot test

The surveys were pilot tested, first by fellow researchers and, secondly, by seven GPs and thereafter revised prior to the survey.

### Statistics

We reported the gender, age and practice type of the responding GPs with the characteristics of GPs in the Region of Southern Denmark as well as with all Danish GPs. We further reported on the descriptive statistics of the different variables.

## Results

A total of 26 GPs registered 1,758 consultations, with an average of 62, ranging from 34–96. Table 1 shows the characteristics of responding GPs, GPs in the Region of Southern Denmark, as well as all GPs in Denmark. The participating GPs were younger than the background population of GPs in the Region of Southern Denmark and GPs in Denmark (Table 1).

**Table 1.** Characteristics of responding GPs, all GPs in the Region of Southern Denmark, and all GPs in Denmark.

	Responding GPs (*N* = 26)	GPs in the Region of Southern Denmark^a^ (*N* = 785)	GPs in Denmark^b^ (*N* = 3365)
Characteristics	*N* (%)	*N* (%)	*N* (%)
Gender			
Female	12 (46.2)	385 (49.0)	1840 (54.7)
Male	14 (53.9)	400 (51.0)	1525 (45.3)
Age			
Below 40	3 (11.5)	50 (6.4)	247 (7.3)
40-49	15 (57.7)	303 (38.6)	1315 (39.1)
50-59	4 (15.4)	232 (29.6)	964 (28.6)
60-69	3 (11.5)	186 (23.7)	776 (23.1)
70 or above	1 (3.9)	14 (1.8)	63 (1.9)
Practice type			
Solo practice	4 (15.4)	148 (18.9)	861 (25.6)
Partnership practice	22 (84.6)	637 (81.1)	2504 (74.4)
Region			
Region of Southern Denmark	26 (100.0)	785 (100.0)	784 (23.3)

^a^Figures from 2017. Source: https://www.laeger.dk/sites/default/files/plo_faktaark_2017_region_syddanmark_0.pdf; ^b^Figures from 2019. Source: https://www.laeger.dk/sites/default/files/laegepopulationen_og_laegepraksispopulationen_2019_0.pdf.

Table 2 includes descriptive statistics on GP, practice and consultation characteristics. A total of 12% of the consultations included at least one defensive medical action.

In 60% of the consultations, patients were female, in 56% of the consultatons, the patient was known in the practice, and in 52% of the consultations, the patient was above 50 years of age. The most common causes for consultations were symptoms from airways and ears (18%), musculoskeletal system (15%), and cardiovascular system (11%) (Table 2).

**Table 2.** Descriptive statistics at consultation level.

Defensive actions	
Defensive action (yes)	12.0% (*n* = 1749)
*GP and practice characteristics*	
GP gender (male)	53.9 % (*n* = 1758)
GP age	Mean = 49.0, Std.Err.= 0.22 (*n* = 1758)
Years as a medical specialist (>12 years)	38.9% (*n* = 1758)
Practice type (solo practice)	13.3% (*n* = 1758)
*Cause of consultation*	
Musculoskeletal	15.4% (*n* = 1746)
Airways and ears	17.9% (*n* = 1746)
Urinary tract, genital organs and pregnancy	10.2% (*n* = 1746)
Psychosocial	10.4% (*n* = 1746)
Skin	9.8% (*n* = 1746)
Cardiovascular system	10.6% (*n* = 1746)
Digestive system	5.1% (*n* = 1746)
Nervous system and eye	3.8% (*n* = 1746)
Endocrine/metabolic	4.9% (*n* = 1746)
Blood and immune system	1.2% (*n* = 1746)
Other causes	6.0% (*n* = 1746)
Several causes	4.7% (*n* = 1746)
*Consultation and patient characteristics*	
Time of the day (forenoon)	65.8% (*n* = 1758)
Patient age (>50 years)	52.7% (*n* = 1758)
Patient gender (male)	40.4% (*n* = 1743)
Previous visits (many)	55.8% (*n* = 1693)

The number of observations vary between variables due to missing values. The coding of the variables is shown in Appendix A.

Table 3 shows the different types of defensive actions, reasons for acting defensively, and assessment of degree of defensiveness at the consultation level. Among the consultations where defensive medical actions took place, the most frequent defensive actions were blood tests (28%), point-of-care-tests (POCT) (20.5%), and referrals (20.0%). The most frequent reasons for acting defensively were reported to be due to: patient influence (37%), concern for overlooking severe disease (32%) and influence from patient relatives (12%). A minority (4%) of the defensive actions were motivated by concern for patient complaint. The GPs characterised a fifth (22%) of the defensive consultations as ‘moderately’ or ‘highly’ defensive (Table 3).

**Table 3.** Types of defensive actions, reasons for acting defensively, and assessment of degree of defensiveness at consultation level.

*Types of defensive actions*	% (*n* = 210)
Point-of-care test	20.5%
Blood test	28.1%
Diagnostic imaging	8.6%
Medicine prescription	15.7%
Referral	20.0%
Hospital admission	2.9%
Referral to rapid diagnostic pathway	1.4%
Record keeping (too long or too much)	10.0%
Appointment about control/follow-up	11.4%
Sick note	1.4%
Other types of actions	2.9%
*Reason for acting defensively*	
Concern for overlooking severe disease	31.9%
Previously overlooked severe disease in patient	1.4%
Concern for patient claim	3.8%
Concern for deviating from professional standard/guideline	8.6%
Influence from other professional	5.2%
Influence from patient	37.1%
Influence from patient relation	12.4%
The patient had a private health insurance	1.0%
Influence from media	1.4%
Time pressure	11.9%
Fatigue	3.3%
Other reasons	6.7%
*Assessment of degree of defensiveness*	
To a great extent defensive	3.3%
To some extent defensive	18.6%
To less extent defensive	68.1%
Not disclosed	10%

A consultation can contain more than one defensive action and there can be several reasons for acting defensively. Therefore, the percentages do not sum to 100% within each category.

## Discussion

Since Denmark essentially has a no-fault malpractice system, it was surprising to find that the GPs in our study reported to carry out DM to the same extent as reported by US and Italian primary care physicians [9,10]. The most frequent defensive action was the ordering of blood tests and POCTs, which may be explained by the fact that these procedures are relatively simple, low-cost procedures, that can be outsourced to the practice nurse. The main reason for DM was reported to stem from influence from the patient, however only seldom due to concern for patient complaints. This is in line with the above applied understanding of DM, where the motive for practicing DM does not relate to malpractice liability as it does in the prevailing international definition of DM where malpractice liability is often mentioned as the primary driver of DM. In interviews with GPs fear of malpractice claims was also not retrieved as a common theme [2]. A fifth of all the defensive actions were characterised as ‘moderately’ or ‘highly’ defensive.

Surprisingly, we found that only 4% of the defensive actions were motivated by concern for claims, but on the other hand influence from patients were the most frequent cause of defensive actions. So why is that? Veldhuis et al. found that deviations from professional standards were often explained by a wish to be nice to patients [14] and a general desire to prevent problems in the doctor-patient relationship [8]. Other reasons may be perceived pressure from strong patients, e.g. well-educated, who are able to (persistently) argue for their wishes, which we found in a previous qualitative focus group study, where GPs argued that strong and demanding patients represent a certain resourceful group of patients who often press the GP into acting defensively [2].

A recent editorial regarding DM [3] pointed out that strong emotional responses to patient complaints can lead to ‘shame’ and desire to withdraw from medical practice due to a feeling of global failure, eventually undermining the physician’s self-esteem [15]. This association is alarming both in the light of the current lack of GPs and the risks of burnout among Danish GPs [16], where the seven-year incidence of burnout has been estimated to 13% [16].

### Strengths

The real-time prospective survey registration of own medical practices in relation to the consultation counteracts recall bias. Moreover, the used survey method is well known by Danish GPs who feel confident with the data management and privacy of their reported data and the whole concept of quality development and supplementary training that is imbedded in the approach [17]. The provided understanding of DM originating from discussions among their peers might have had the positive effect that the participating GPs have been able to relate to this particular understanding of DM.

### Limitations

The study had a rather low participation rate and it may be assumed that the GPs participating in our study may have had a special interest in the subject, and therefore the focus on own defensive actions may overestimate the occurrence of DM. The low number of participating GPs makes the findings related to GP characteristics susceptible to sampling bias. Specifically, the participating GPs were younger than the background population of GPs in the surveyed region.

It could be assumed that the GPs may have increased the awareness of their own defensive behaviour during the observation period. Therefore, our results may overestimate the occurrence of DM. Moreover, divergent understandings of the nature of DM amongst the GPs cannot be ruled out even though they were provided with a pre-formulated definition of DM. The individual GPs’ perception of acting defensively may to some extent mirror his/her risk behaviour, which may again be related to his/her experiences with malpractice and claims. However, only a minority of the defensive actions were motivated by fear for claims.

Our results may be generalizable to GPs in countries with comparable health – and complaint systems, but not to countries with a more widespread malpractice litigation.

We acknowledge that the individual situation and the individual GP’s perception of the situation, and her/his actions, is decisive for the assessment of whether the action can be characterized as defensive or not. Therefore, we expect that some actions may have been defensive according to one GP, but not to another.

### Perspectives

Ideally, all physicians, across specialties and sectors, should collaborate by discussing and sharing best patient care [18], for example, radiologists could help educate referring colleagues about the benefits and risks of diagnostic imaging prescribed [19]. A climate of clinical collaboration would be conducive to the best use of healthcare services [12], eventually reducing DM costs [20]. Moreover, it should be recognised that medicine is not an exact science [21,22] as certainty is non-existent in medical practice [23]. Patients’ trust in physicians has decreased in the last decades, maybe because physicians’ time spent with the individual patient has been reduced [24]. If patients do not trust their physician, they will become reluctant to provide important information [4]. Trust in the physician–patient relationship should be further re-enforced as this may be the most efficient antidote to DM [1].

Future research should explore the societal and personal mechanisms, including physicians’ risk perceptions and risk adversions, underlying DM, as well as the consequences of DM in order to provide realistic and secure health care solutions for both patients and health care professionals.

## Conclusion

Danish GPs registered self-perceived defensive actions in a prospective survey. DM was carried out in one out of eight consultations, most often due to patient influence. The most frequent defensive actions were blood tests, POCTs and referrals.

## Data Availability

The study has been approved by the legal services SDU RIO (SDU Research & Innovation Organisation) managing the interests of the Danish Data Protection Agency, project number 10.321.

## Appendix A.

### Coding of variables

**Table:**

*Defensive action*
Defensive action
‘Yes’ = 1
‘No’ = 0
*GP and practice characteristics*
GP gender
‘Male’ = 1
‘Female’ = 0
GP age
Treated as a continuous variable
Years as a medical specialist
‘More than 12 years of experience’ = 1
’12 years of experience or less’ = 0
Practice type
‘Single handed practice’ = 1
‘Partnership practice’ = 0
*Consultation and patient characteristics*
Time of the day
Before 12 o’clock = 1
12 o’clock or later = 0
Patient age
Patient over 50 years of age = 1
Patient 50 years of age or less = 0
Patient gender
‘Male’ = 1
‘Female’ = 0
Previous visits
‘Many previous consultations’ = 1
‘A few previous consultations’, ‘No previous consultations’ = 0
*Cause of consultation*
Musculoskeletal
‘Musculoskeletal = 1
‘Other causes’ = 0
Airways and ears
‘Airways including ears’ = 1
‘Other causes’ = 0
Urinary tract, genital organs and pregnancy
‘Urinary tract, genital organs and pregnancy’= 1
‘Other causes’ = 0
Psychosocial
‘Psychosocial’= 1
‘Other causes’ = 0
Skin
‘Skin’ = 1
‘Other causes’ = 0
Cardiovascular system
‘Cardiovascular’= 1
‘Other causes’ = 0
Digestive system
‘Digestion’ = 1
‘Other causes’ = 0
Nervous system and eye
‘Nervous system and eye’= 1
‘Other causes’ = 0
Endocrine/metabolic
‘Endocrine/metabolic’= 1
‘Other causes’ = 0
Blood and immune system
‘Blood and immune system’= 1
‘Other causes’ = 0
Other causes
‘Other causes’ = 1
‘Other causes (than other causes)’ = 0
Several causes
‘Several causes’ = 1
‘Single causes’ = 0
